# Joint User Scheduling, Relay Selection, and Power Allocation for Multi-Antenna Opportunistic Beamforming Systems

**DOI:** 10.3390/e23101278

**Published:** 2021-09-29

**Authors:** Wenbin Sun, Mingliang Tao, Xin Yang, Tao Zhang, Chuang Han, Ling Wang

**Affiliations:** 1 School of Electronics and Information, Northwestern Polytechnical University, Xi’an 710072, China; sunwenbin@nwpu.edu.cn (W.S.); hanchuang@nwpu.edu.cn (C.H.); lingwang@nwpu.edu.cn (L.W.); 2 China Academy of Launch Vehicle Technology, Beijing 100076, China; zhangtao.nankai@163.com

**Keywords:** multi-antenna, opportunistic beamforming, relay, resource scheduling

## Abstract

Opportunistic beamforming (OBF) is a potential technique in the fifth generation (5G) and beyond 5G (B5G) that can boost the performance of communication systems and encourage high user quality of service (QoS) through multi-user selection gain. However, the achievable rate tends to be saturated with the increased number of users, when the number of users is large. To further improve the achievable rate, we proposed a multi-antenna opportunistic beamforming-based relay (MOBR) system, which can achieve both multi-user and multi-relay selection gains. Then, an optimization problem is formulated to maximize the achievable rate. Nevertheless, the optimization problem is a non-deterministic polynomial (NP)-hard problem, and it is difficult to obtain an optimal solution. In order to solve the proposed optimization problem, we divide it into two suboptimal issues and apply a joint iterative algorithm to consider both the suboptimal issues. Our simulation results indicate that the proposed system achieved a higher achievable rate than the conventional OBF systems and outperformed other beamforming schemes with low feedback information.

## 1. Introduction

Multiple-input-multiple-output (MIMO) is one of the key techniques for the fifth generation (5G) and beyond 5G (B5G) [1,2,3] that can improve the system performance through multi-antenna technique without other extra wireless resource. Due to the high performance, many studies have been conducted on MIMO, for example, beamforming, spatial modulation, millimeter wave (mmWave), and multiple access.

A compressed sensing (CS)-based orthogonal matching pursuit (OMP) detection is proposed in [4], which achieved a satisfactory performance with a low computational complexity. Then, an equalizer was designed to mitigate the multiuser interference and improve the detection performance. In [5], the authors focused on the associated massive backhaul traffic of high dense heterogeneous networks (HetNets). Backhaul traffic caused by heavy signalling in mmWave-based 5G HetNets was reduced through a cluster-based central architecture.

The authors proposed a novel radio access network-level, HetNet, in [6], which can efficiently serve the small cells under long term evolution (LTE). In the proposed scheme, Wi-Fi is applied as a bridge to connect the large and small coverage regions. A two-level control and user data planes splitting is also provided to efficiently utilize band and decrease the complexity of HetNet.

For most of the research, complete channel state information (CSI) is required to preprocess the transmit signals. When complete CSI is unavailable at base station (BS), the system performance descends rapidly [7]. In order to deal with the performance decline, opportunistic beamforming (OBF) was proposed [8].

OBF is a kind of transmit preprocessing scheme, where random weights rather than the designed weights are multiplied by the transmit signals. Due to random weights, users are not required to feed complete CSI back to BS, which leads to low feedback information and complexity. Ref. [9] indicates two transmission mechanisms for OBF systems in detail, including the max-capacity and fairness mechanisms. Based on these two mechanisms, the closed-form expressions of capacities and the bounds of bit error ratios (BERs) are derived.

In [10], the authors proposed a multi-weight scheme for OBF, where multiple variable weights were introduced to improve the selection gain. Then, the optimal numbers of weights were discussed according to different conditions. To further reduce the feedback bits, the signal-to-noise ratio (SNR) quantization method was proposed in [11]. The quantization levels were derived according to the received SNRs.

The authors in [12] indicated the optimal feedback thresholds based on the user number and channel characteristics. Moreover, in [13,14], the multiple access schemes of OBF are presented, including orthogonal multiple access (OMA) and non-orthogonal multiple access (NOMA) schemes. Compared to the OMA scheme, the NOMA scheme achieves a larger sum rate, while the complexity of the receiver is higher.

MmWave is a potential MIMO approach that can be applied in OBF to achieve high performance without extra wireless resources. Many works have been done on MmWave. In [15], the authors proposed an adaptive mmWave beamforming training method with compressive sensing based channel estimation. With respect to the statistics of the angular spread, both the angles of departures and arrivals were estimated according to the position of mobile station. Considering a mmWave device-to-device (D2D) communications scenario, an interference cancellation is presented based on the users’ locations in [16].

Both localization and compressive sensing techniques are utilized to detect the best pair beams between the transmitter and receiver. According to the numerical results, the proposed scheme provided higher spectral efficiency and energy efficiency than the conventional D2D approach. Due to both the highly dynamic time-variant channel and high blocking probability, it is necessary to apply an efficient resource scheduling with considering the channel changes for the maximum system performance. Therefore, in [17], a proportional fairness-based channel sensitive scheduling scheme was introduced in the downlink mmWave network to achieve a balance between the total system rate and fairness for users.

However, when the number of user is large, the performances of OBF systems reach saturation points. To deal with the saturation and further improve performance, many works have been done in [18,19,20,21]. Relay is one of the most effective techniques. In a relay-based system, both relay and BS serve users through a cooperative method, which enhances the received SNR and improves the system performance [22]. Due to the performance improvement, some researchers focused on introducing relay into OBF systems.

The authors proposed a cooperative diversity OBF scheme [23], which applies a relay node to improve the performance of a distant destination receive node. In [24], a two-way opportunistic multiuser relay system was presented, and then a pair scheduling algorithm based on channel aligning was proposed to reduce the inter-pair interference between the users. However, for both the previous works, only one relay node was considered, and the multi-relay selection strategy was neglected. Moreover, the authors ignored the direct channel between the BS and user, which is not appropriate for physical systems.

This paper proposes a multi-antenna opportunistic beamforming-based relay (MOBR) system that expands one relay to multiple relays. Both relay and direct channels are considered in the proposed system. To maximize the achievable rate of MOBR system, we formulate an optimization problem. However, the optimization problem is a non-deterministic polynomial (NP)-hard problem. Due to the difficulty of solving the NP-hard problem, we divide the origin optimization problem into two suboptimal issues and apply a joint iterative algorithm to obtain the solution of the optimization problem. The contributions of this paper can be summarized in the following three aspects:

1. We present a downlink MOBR system in Rayleigh fading channels, which can obtain both multi-relay and multi-user selection gains.
2. The influence of direct channel between BS and user is taken into consideration. Then, an optimization problem is proposed to maximize the achievable rate.
3. The optimization problem is decomposed into two suboptimal issues. Based on the analyses of the two suboptimal issues, a joint iterative algorithm is used to achieve the solution of the original optimization problem.

This paper is organized as follows. The system model of MOBR is introduced in Section 2. Section 3 formulates an optimization problem to obtain the maximum achievable rate. In Section 4, we reformulate the original problem and propose a joint iterative algorithm. Our simulation results are shown in Section 5. Finally, our conclusions are summarized in Section 6.

## 2. System Model

During this paper, let *x* and x present a variable and a vector, respectively. $x^{T}$ indicates the transpose. ∥·∥ denotes the Frobenius norm of a vector, and |·| represents the absolute value of a variable. C is applied to denote the complex space. O(·) represents the infinitesimal of higher order. The meanings of the notations are listed in Appendix A.

**Figure 1:**
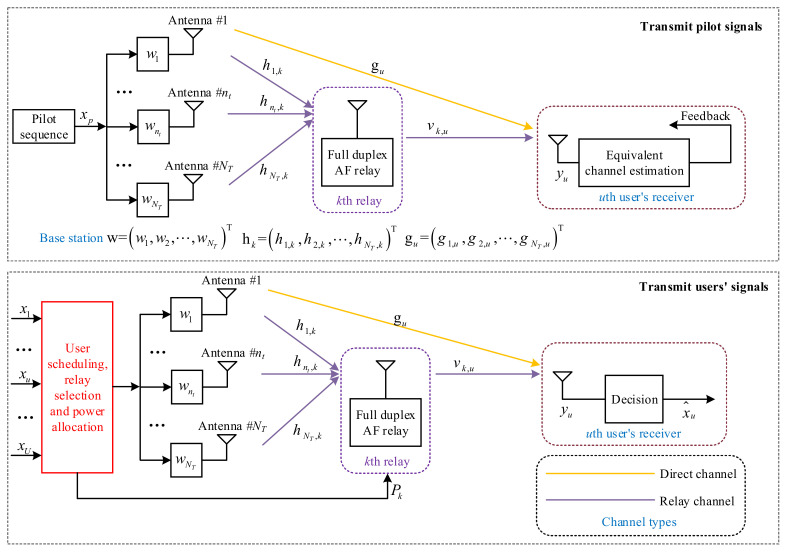
Multi-antenna opportunistic beamforming-based relay system model.

The system model of MOBR is presented in Figure 1. There are a BS, *K* (K≥2) amplify-and-forward (AF) relays, and *U* (U≥2) users in a MOBR system, where the received signals are directly amplified and forwarded in relay nodes. $N_{T}$ $\left(N_{T}\geq 2\right)$ transmit antennas are equipped at the BS. Both pilot and users’ signals are transmitted by the BS. *U* users are randomly located at different places, and only one receiving antenna is equipped at each user. *K* relays are applied to improve the users’ performance, and each relay has one antenna. For simplicity, we consider full duplex mode for each relay, and ignore the interferences among the relays.

A set of random coefficients, denoted by $w_{1},w_{2},\cdots ,w_{n_{t}},\cdots ,w_{N_{T}}$, $\left(1\leq n_{t}\leq N_{T}\right)$, is applied to preprocess the transmit signals. The random coefficient $w_{n_{t}}$ is given by

(1) $$w_{n_{t}}=\sqrt{\alpha_{n_{t}}}e^{j\phi_{n_{t}}},$$

where $\sqrt{\alpha_{n_{t}}}$ and $\phi_{n_{t}}$ denote the amplitude and phase, respectively. *j* represents the symbol of imaginary number. The vector form of the random coefficients is

(2) $$w={\left[w_{1},w_{2},\cdots ,w_{n_{t}},\cdots ,w_{N_{T}}\right]}^{T},$$

where $w\in C^{N_{T}\times 1}$. To guarantee the power constraint, we set

(3) $${∥w∥}^{2}=\sum_{n_{t}=1}^{N_{T}}{\left|w_{n_{t}}\right|}^{2}=\sum_{n_{t}=1}^{N_{T}}{\left|\sqrt{\alpha_{n_{t}}}e^{j\phi_{n_{t}}}\right|}^{2}=1.$$

Users can receive signals from both direct and relay channels. The relay channel contains the channels from the BS to the relay and from the relay to the user. Let $g_{u}\in C^{N_{T}\times 1}$, $h_{k}\in C^{N_{T}\times 1}$ and $v_{k,u}\in C^{1\times 1}$ denote the channels from the BS to the *u*th user, from the BS to the *k*th relay and from the *k*th relay to the *u*th user, respectively.

$g_{u}$ is given by

(4) $$g_{u}={\left[g_{1,u},g_{2,u},\cdots ,g_{n_{t},u},\cdots ,g_{N_{T},u}\right]}^{T},$$

where $g_{n_{t},u}$ represents the channel coefficient between the $n_{t}$th transmit antenna of the BS and the *u*th user’s receiving antenna.

$h_{k}$ can be expressed as

(5) $$h_{k}={\left[h_{1,k},h_{2,k},\cdots ,h_{n_{t},k},\cdots ,h_{N_{T},k}\right]}^{T},$$

where $h_{n_{t},k}$ is the channel coefficient between the $n_{t}$th transmit antenna of the BS and the receiving antenna of the *k*th relay.

A quasi-static scenario is considered, where $g_{u}$, $h_{k}$ and $v_{k,u}$ remain unchanged during the coherent time. The mathematic models of $g_{u}$, $h_{k}$ and $v_{k,u}$ are given by circular symmetric complex Gaussian random variables with zero mean and unit variance, which is represented as CN (0,1).

To evaluate direct and relay channels, pilot sequences are transmitted, which occupy two symbols. We denote pilot sequences by $x_{p,1}$ and $x_{p,2}$, and they satisfy $E\left[x_{p,1}x_{p,1}^{*}\right]=E\left[x_{p,2}x_{p,2}^{*}\right]=1$.

Evaluate direct channel: Set the amplified power of the relay $P_{k}=0$, and the received signal of the user is

(6) $$y_{p,1}=g_{u}^{T}wx_{p,1}+z_{u}=\sum_{n_{t}=1}^{N_{T}}g_{n_{t},u}w_{n_{t}}x_{p,1}+z_{u},$$

where $z_{u}$ represents additive white Gaussian noise (AWGN) with variance $\sigma_{z_{u}}^{2}$. We denote the equivalent direct channel by

(7) $$I_{u}^{d}=g_{u}^{T}w=\sum_{n_{t}=1}^{N_{T}}g_{n_{t},u}w_{n_{t}}.$$

Since $x_{p,1}$ is a known pilot sequence, $I_{u}^{d}$ can be obtained by the users. Then, each user feeds its respective equivalent direct channel $I_{u}^{d}$ back to the BS. Evaluate relay channel: Transmit the pilot sequence $x_{p,2}$, and set the amplified power of the relay $P_{k}=P_{0}\neq 0$. Thus, the received signal of the user includes direct and relay signals, which is given by

(8) $$y_{p,2}=v_{k,u}\sqrt{P_{0}}\left(h_{k}^{T}wx_{p,2}+z_{k}\right)+g_{u}^{T}wx_{p,2}+z_{u}.$$

$z_{u}$ denotes AWGN with variance $\sigma_{z_{k}}^{2}$. Since both $x_{p,2}$ and $g_{u}^{T}w$ are known by the users, the direct signals can be removed from $y_{p,2}$.

After removing the direct signals, the received signal becomes

(9) $$\begin{array}{cc} {\bar{y}}_{p,2} & =v_{k,u}\sqrt{P_{0}}\left(h_{k}^{T}wx_{p,2}+z_{k}\right)+z_{u}\\ \; & =\sqrt{P_{0}}v_{k,u}h_{k}^{T}wx_{p,2}+\left(\sqrt{P_{0}}v_{k,u}z_{k}+z_{u}\right). \end{array}$$

We denote the equivalent relay channel by

(10) $$I_{u}^{r}=v_{k,u}h_{k}^{T}w=v_{k,u}\sum_{n_{t}=1}^{N_{T}}h_{n_{t},k}w_{n_{t}}.$$

Similar to the equivalent direct channel $I_{u}^{d}$, the equivalent relay channel $I_{u}^{r}$ can also be obtained by users, and the users feed $I_{u}^{r}$ back to the BS. Users are only required to return both the equivalent direct and relay channels rather than complete CSI in MOBR systems, which can significantly reduce the number of feedback bits and the complexity of the feedback link.

After receiving the feedback equivalent channels from the users, the BS applies user scheduling, relay selection, and power allocation to transmit the users’ signals. The received signal of the *u*th user is

(11) $$\begin{array}{cc} y_{u} & =v_{k,u}\sqrt{P_{k}}\left(h_{k}^{T}w\sqrt{P_{b}}x_{u}+z_{k}\right)+g_{u}^{T}w\sqrt{P_{b}}x_{u}+z_{u}\\ \; & =\sqrt{P_{k}}\sqrt{P_{b}}I_{u}^{r}x_{u}+\sqrt{P_{b}}I_{u}^{d}x_{u}+\left(v_{k,u}\sqrt{P_{k}}z_{k}+z_{u}\right), \end{array}$$

where $P_{b}$ is the allocated power of the BS.

## 3. Problem Formulation

To maximize the rates of the users, an optimization problem is formulated in this section.

### 3.1. Objective Function

According to (11), the received SNR of the *u*th user is

(12) $$\gamma_{u}=\frac{{\left|\sqrt{P_{k}}\sqrt{P_{b}}I_{u}^{r}+\sqrt{P_{b}}I_{u}^{d}\right|}^{2}}{P_{k}v_{k,u}^{2}\sigma_{z_{k}}^{2}+\sigma_{z_{u}}^{2}}.$$

Since $v_{k,u}∼\mathrm{CN}\left(0,1\right)$, $\gamma_{u}$ can be approximately expressed as

(13) $$\gamma_{u}\approx \frac{{\left|\sqrt{P_{k}}\sqrt{P_{b}}I_{u}^{r}+\sqrt{P_{b}}I_{u}^{d}\right|}^{2}}{P_{k}\sigma_{z_{k}}^{2}+\sigma_{z_{u}}^{2}}.$$

Thus, the achievable rate per unit bandwidth of MOBR is given by

(14) $$R=\log_{2}\left(1+\gamma_{u}\right)\approx \log_{2}\left(1+\frac{{\left|\sqrt{P_{k}}\sqrt{P_{b}}I_{u}^{r}+\sqrt{P_{b}}I_{u}^{d}\right|}^{2}}{P_{k}\sigma_{z_{k}}^{2}+\sigma_{z_{u}}^{2}}\right).$$

During the rest of this paper, we apply “the achievable rate” to represent “the achievable rate per unit bandwidth” for simplification.

### 3.2. Constraints

Total power constraint: We consider a power limited system, where the total power is constant. Thus, we have

(15) $$C1:P_{b}+P_{k}=P_{\mathrm{total}}.$$

BS and relay power constraints: The allocated power of both BS and relay is positive. Moreover, the power of relay is smaller than a constant $P_{k}^{\max}$, due to the limited volume and power of relay. The BS and relay power constraints can be given by

(16) $$C2:P_{b}\geq 0.$$

(17) $$C3:0\leq P_{k}\leq P_{k}^{\max}.$$

Relay set constraint: There are *K* relays in a MOBR system, and the set of relays is denoted by $K_{r}$. The BS selects a relay from the set $K_{r}$ to collaborate; thus, the relay set constraint is expressed as

(18) $$C4:k\in K_{r}.$$

User set constraint: *U* users are involving in a MOBR system, and the user set is defined by $U_{u}$. The BS schedules a user from $U_{u}$ to transmit signal according to the feedback information. User set constraint can be written as

(19) $$C5:u\in U_{u}.$$

### 3.3. Optimization Problem

Based on the previous analyses, an optimization problem to maximize the achievable rate can be formulated as

(20) $$\begin{array}{cc} \; & \max_{P_{b},P_{k},k,u}R=\log_{2}\left(1+\frac{{\left|\sqrt{P_{k}}\sqrt{P_{b}}I_{u}^{r}+\sqrt{P_{b}}I_{u}^{d}\right|}^{2}}{P_{k}\sigma_{z_{k}}^{2}+\sigma_{z_{u}}^{2}}\right),\\ \; & s.t.\;C1-C5. \end{array}$$

We found that the proposed optimization problem (20) is a NP-hard problem and is difficult to solve due to the following reasons:

(1) Both $P_{b}$ and $P_{k}$ are continuous variables; both *k* and *u* are discrete integer variables. Therefore, the feasible set of problem (20) is non-convex.

(2) Objective function is also non-convex, since logarithmic, polynomial, and fractional subfunctions are included in the objective function.

## 4. Problem Reformulation and Solution

Since the logarithmic function is monotonic, the original optimization problem (20) can be equivalently rewritten as

(21) $$\begin{array}{cc} \; & \max_{P_{b},P_{k},k,u}D=\frac{{\left|\sqrt{P_{k}}\sqrt{P_{b}}I_{u}^{r}+\sqrt{P_{b}}I_{u}^{d}\right|}^{2}}{P_{k}\sigma_{z_{k}}^{2}+\sigma_{z_{u}}^{2}},\\ \; & s.t.\;C1-C5. \end{array}$$

To solve the optimization problem (21), two suboptimal issues are proposed, and then a joint iterative algorithm is applied to consider both the suboptimal issues.

### 4.1. Power Allocation

First, we consider power allocation with the given *k* and *u*. We denote η as a parameter belonging to (0,1), and set $P_{k}=\eta P_{\mathrm{total}}$ and $P_{b}=\left(1-\eta \right)P_{\mathrm{total}}$. Thus, the optimization problem (21) can be simplified as

(22) $$\begin{array}{cc} \max_{\eta }D_{1}= & \frac{{\left[\sqrt{\eta P_{\mathrm{total}}}\sqrt{\left(1-\eta \right)P_{\mathrm{total}}}I_{u}^{r}+\sqrt{\left(1-\eta \right)P_{\mathrm{total}}}I_{u}^{d}\right]}^{2}}{\eta P_{\mathrm{total}}\sigma_{z_{k}}^{2}+\sigma_{z_{u}}^{2}},\\ s.t.\; & C6.\;0\leq \eta \leq 1,\\ \; & C7.\;\eta \leq \frac{P_{k}^{\max}}{P_{\mathrm{total}}}. \end{array}$$

The suboptimal problem (22) is also difficult to solve, due to the complex mathematical expressions. To simplify the mathematical expressions, we suppose $\vartheta_{1}=P_{\mathrm{total}}I_{u}^{r}$, $\vartheta_{2}=\sqrt{P_{\mathrm{total}}}I_{u}^{d}$ and $\vartheta_{3}=P_{\mathrm{total}}\sigma_{z_{k}}^{2}$. Moreover, since $\sigma_{z_{u}}^{2}$ is a constant, the objective function of (22) can be expressed as

(23) $$\begin{array}{cc} \; & \max_{\eta }\frac{{\left[\sqrt{\eta P_{\mathrm{total}}}\sqrt{\left(1-\eta \right)P_{\mathrm{total}}}I_{u}^{r}+\sqrt{\left(1-\eta \right)P_{\mathrm{total}}}I_{u}^{d}\right]}^{2}}{\eta P_{\mathrm{total}}\sigma_{z_{k}}^{2}+\sigma_{z_{u}}^{2}}\\ \Leftrightarrow \; & \max_{\eta }\frac{{\left[\vartheta_{1}\sqrt{\eta }\sqrt{1-\eta }+\vartheta_{2}\sqrt{1-\eta }\right]}^{2}}{\vartheta_{3}\eta }\\ \Leftrightarrow \; & \max_{\sqrt{\eta }}\frac{\vartheta_{1}\sqrt{\eta }\sqrt{1-\eta }+\vartheta_{2}\sqrt{1-\eta }}{\sqrt{\vartheta_{3}}\sqrt{\eta }}. \end{array}$$

Let $\lambda \overset{\Delta }{\;=}\sqrt{\eta }$, and the suboptimal issue (22) can be transformed as

(24) $$\begin{array}{cc} \; & \max_{\lambda }{\tilde{D}}_{1}=\frac{\vartheta_{1}\lambda \sqrt{1-\lambda^{2}}+\vartheta_{2}\sqrt{1-\lambda^{2}}}{\sqrt{\vartheta_{3}}\lambda },\\ s.t.\; & C8.\;0\leq \lambda \leq \sqrt{\frac{P_{k}^{\max}}{P_{\mathrm{total}}}}. \end{array}$$

To obtain the solution of (24), we solve the stationary points of ${\tilde{D}}_{1}$ and then compare the function values of the stationary points with the feasible region endpoints.

With respect to η, the derivative of ${\tilde{D}}_{1}$ is given by

(25) $$\frac{d{\tilde{D}}_{1}}{d\lambda }=\frac{\vartheta_{1}\sqrt{1-\lambda^{2}}-\frac{\vartheta_{1}\lambda^{2}+\vartheta_{2}\lambda }{\sqrt{1-\lambda^{2}}}}{\sqrt{\vartheta_{3}}\lambda }-\frac{\vartheta_{1}\lambda \sqrt{1-\lambda^{2}}+\vartheta_{2}\sqrt{1-\lambda^{2}}}{\sqrt{\vartheta_{3}}\lambda^{2}}.$$

Let $\frac{d{\tilde{D}}_{1}}{d\eta }=0$, and we can obtain the stationary point of ${\tilde{D}}_{1}$ as

(26) $$\lambda_{sp}=-{\left(\frac{\vartheta_{2}}{\vartheta_{1}}\right)}^{\frac{1}{3}}.$$

Based on both C8 and C9, the feasible region of λ is given by

(27) $$\begin{array}{cc} S_{\lambda } & =[s_{\lambda ,\mathrm{left}},s_{\lambda ,\mathrm{right}}]\\ \; & =\left[\max\left\{-1,-\sqrt{\frac{P_{k}^{\max}}{P_{\mathrm{total}}}}\right\},\min\left\{1,\sqrt{\frac{P_{k}^{\max}}{P_{\mathrm{total}}}}\right\}\right]. \end{array}$$

The optimal λ is achieved as

(28) $$\lambda_{opt}=\underset{\lambda_{sp},s_{\lambda ,\mathrm{left}},s_{\lambda ,\mathrm{right}}}{\arg}\max{\tilde{D}}_{1}\left(\lambda \right).$$

Hence, the optimal parameter $\eta_{opt}$ satisfies $\eta_{opt}=\lambda_{opt}^{2}$, and then the power of the BS and relay are $P_{b}=\eta_{opt}P_{\mathrm{total}}$ and $P_{k}=\left(1-\eta_{opt}\right)P_{\mathrm{total}}$, respectively.

### 4.2. User Scheduling and Relay Selection

This subsection discusses the assumption that the power allocation result has been obtained. The BS selects both the optimal relay and user to transmit signals. Thus, the optimization problem (21) can be transformed as

(29) $$\begin{array}{cc} \max_{I_{u}^{r},I_{u}^{d}}D_{2}= & \frac{\sqrt{\eta P_{\mathrm{total}}}\sqrt{\left(1-\eta \right)P_{\mathrm{total}}}I_{u}^{r}+\sqrt{\left(1-\eta \right)P_{\mathrm{total}}}I_{u}^{d}}{\sqrt{\eta P_{\mathrm{total}}\sigma_{z_{k}}^{2}+\sigma_{z_{u}}^{2}}},\\ s.t.\; & C9.\;k\in K_{r},\\ s.t.\; & C10.\;u\in U_{u}. \end{array}$$

Our object is to find the suitable relay and user to maximize the achievable rate. It is noted that both *k* and *u* are discrete and selected from the finite sets. Therefore, the optimal solutions can be obtained through a global exhaustive searching algorithm. The global exhaustive searching number equals to K×U. With the obtained optimal *k*, *u* and power allocation result, we can calculate the maximum achievable rate according to (14).

### 4.3. A Joint Iterative Algorithm

Based on both the previous suboptimal issues, we propose an iterative algorithm, which jointly considers user scheduling, relay selection, and power allocation. The details of the proposed algorithm are listed in Algorithm 1.
**Algorithm 1** A joint iterative algorithm.  1:**Input:***K*, *U*, $I_{u}^{r}$, $I_{u}^{d}$, $\sigma_{z_{k}}^{2}$, $\sigma_{z_{u}}^{2}$, $K_{r}$, $U_{u}$, $P_{\mathrm{total}}$ and $P_{k}^{\max}$.  2:**Output:** The maximum achievable rate and corresponding *k*, *u*, $P_{b}$ and $P_{k}$.  3:Initialize: $k_{i_{1}}=1$, $u_{i_{2}}=1$, $P_{k}=P_{k}^{\max}$ and $P_{b}=P_{\mathrm{total}}-P_{k}^{\max}$, where $i_{1}$ and $i_{2}$ are respectively the labels of relay and user;  4:**while**$\;k_{i_{1}}\in [1,K]\;$**do**  5:  **while** $\;u_{i_{2}}\in \left[1,U\right]\;$ **do**  6:    $k=k_{i_{1}}$;  7:    $u=u_{i_{2}}$;  8:    $I_{u}^{r}=I_{u_{i_{2}}}^{r}$;  9:    $I_{u}^{d}=I_{u_{i_{2}}}^{d}$;10:    Allocate the power for both BS and relay based on (22);11:    Calculate the achievable rate through (14);12:    Record *R*, *k*, *u*, $P_{b}$ and $P_{k}$;13:     $k_{i_{1}}=k_{i_{1}}+1$;14:     $u_{i_{2}}=u_{i_{2}}+1$;15:   **end while**16:**end while**17:Search the maximum achievable rate *R* and corresponding relay *k*, user *u* and power allocation result $P_{b}$ and $P_{k}$.

According to the proposed Algorithm 1, we decided on all of the parameters, including the cooperative relay *k*, the user *k*, the power of BS $P_{b}$ and the power of relay $P_{k}$. Then, the signal of the *u*th user is transmitted after OBF preprocessing through a cooperative method with the *k*th relay.

Convergence: The convergence of Algorithm 1 is determined by the convergence of the two suboptimal issues, i.e., power allocation and user scheduling and relay selection. For power allocation issue, the suboptimal issue is convergent, since the solution is obtained through a stationary point method and (25) has a solution. Moreover, considering the user scheduling and relay selection issue, a global exhaustive searching algorithm is applied to obtain the solutions. The number of searching is K×U, which leads to the convergence of the user scheduling and relay selection issue. Thus, Algorithm 1 is convergent.

Complexity: The complexity of Algorithm 1 are composed of the complexities of both power allocation and user scheduling and relay selection issues. The complexity of power allocation with a stationary point method is $O\left(\zeta_{p}\right)$, where $\zeta_{p}$ represents the complexity to complete once stationary point method [25]. The complexity of user scheduling and relay selection issue is determined by the number of exhaustive searching and equals to $O\left(K\times U\right)$. Here, the complexity of Algorithm 1 is expressed as $O(\zeta_{p}\times K\times U)$.

## 5. Numerical Results

In this section, we evaluate the performance of MOBR systems in Rayleigh fading channels. During the simulations, we apply binary phase shift keying (BPSK) modulation. The details of the simulation parameters are listed in Table 1.

**Figure 2:**
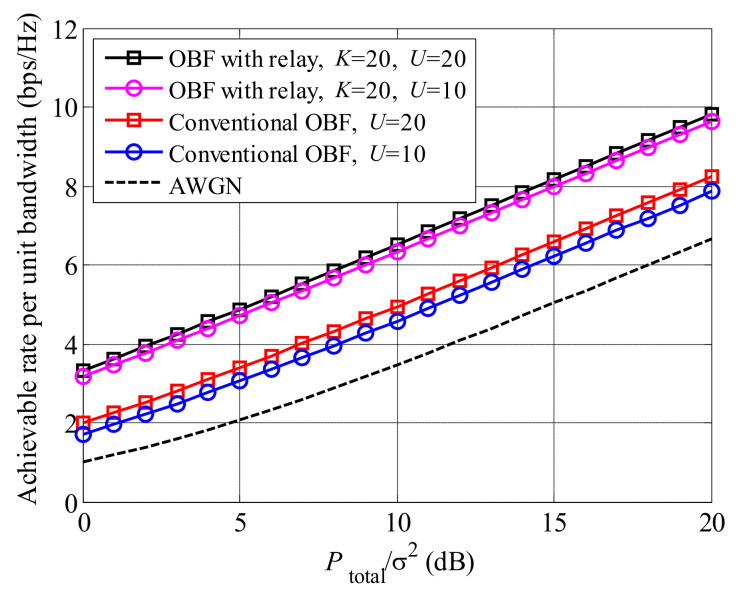
Comparison between MOBR and the conventional OBF scheme.

Figure 2 presents the achievable rate of MOBR and the conventional OBF without relay. All curves raise with the increased $\frac{P_{\mathrm{total}}}{\sigma^{2}}$. Comparing the curves between MOBR and the conventional OBF systems, we found that the relay scheme can significantly improve the achievable rate of an OBF system. The reason is that relays enhance the received SNRs through the cooperative transmission method and multi-relay selection. Furthermore, all curves are higher than the AWGN case, due to multi-user and multi-relay selection gains.

**Figure 3:**
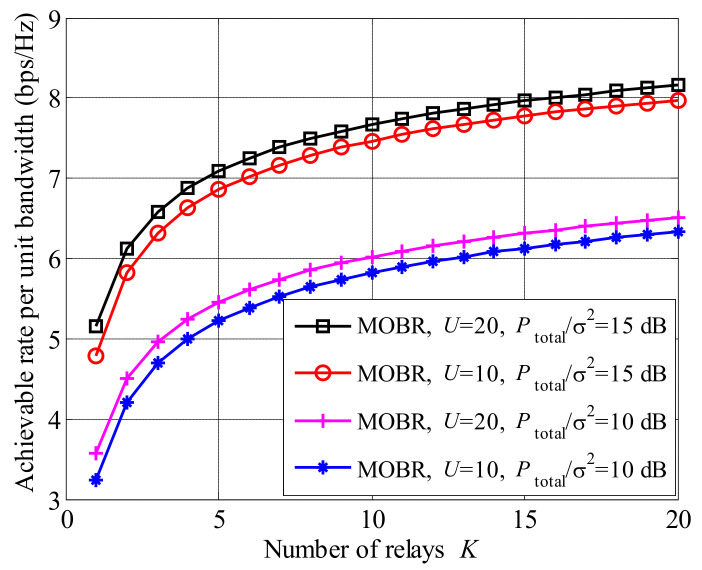
Achievable rate versus the number of relays *K*.

In Figure 3, we show the relationship between the achievable rate and the number of relays *K*. We found that the achievable rates of MOBR were greatly improved with the increased number of relays *K*. For example, when K=1, U=20 and $\frac{P_{\mathrm{total}}}{\sigma^{2}}=15$ dB, the achievable rate approximately equals 5.2 (bps/Hz), while, when K=20, the achievable rate goes up to 8.2 (bps/Hz). The gain of the achievable rate comes from collaboration and multi-relay selection. Moreover, larger *U* and $\frac{P_{\mathrm{total}}}{\sigma^{2}}$ lead to higher achievable rates.

**Figure 4:**
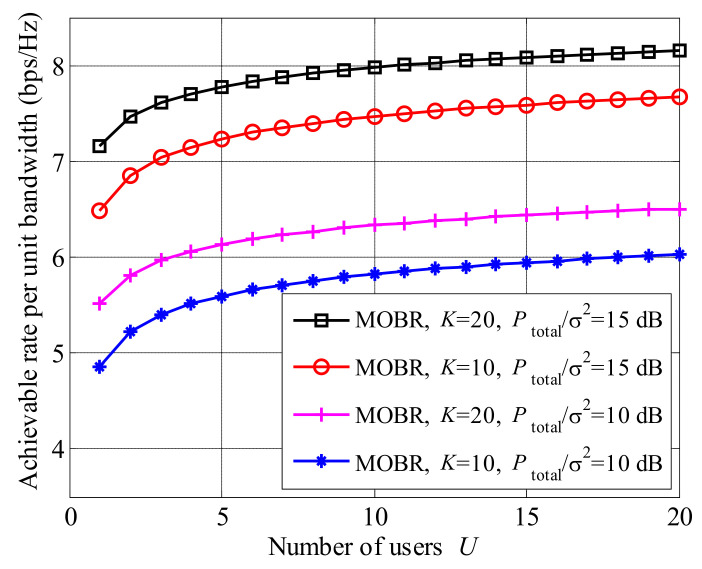
Achievable rate with the increased number of users *U*.

To illustrate the influence of the user number *U*, we plot the achievable rate versus the number of users in Figure 4. The achievable rate of MOBR improves as the number of users increases. Moreover, compared to Figure 3, the growth of the achievable rate with the number of users *U* is less than that with the number of relays *K*. When U≥20, the curves are almost stable. The reason is that the gain from multi-user selection approximates lnU, which is stable with a large *U*.

**Figure 5:**
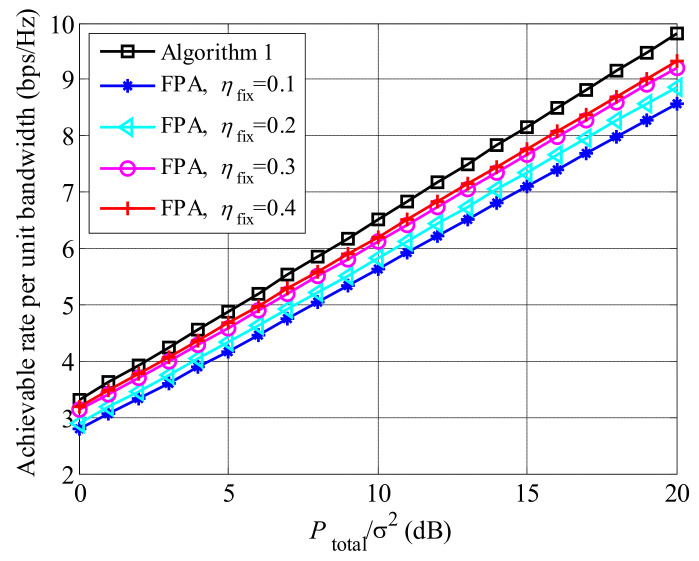
The comparison of achievable rates between Algorithm 1 and FPA, where K=20 and U=20.

We compare the achievable rates between Algorithm 1 and fixed power algorithm (FPA) [27] in Figure 5, where K=20 and U=20. For FPA scheme, users and relays are scheduled based on (29), and $P_{k}=\eta_{\mathrm{fix}}P_{\mathrm{total}}$ and $P_{b}=\left(1-\eta_{\mathrm{fix}}\right)P_{\mathrm{total}}$. It is seen that Algorithm 1 obtains higher achievable rate than the FPA scheme, no matter of the value of $\eta_{\mathrm{fix}}$, since the proposed algorithm can adaptively adjust the power allocation to maximize the achievable rate according to the equivalent channels $I_{u}^{r}$ and $I_{u}^{d}$.

**Figure 6:**
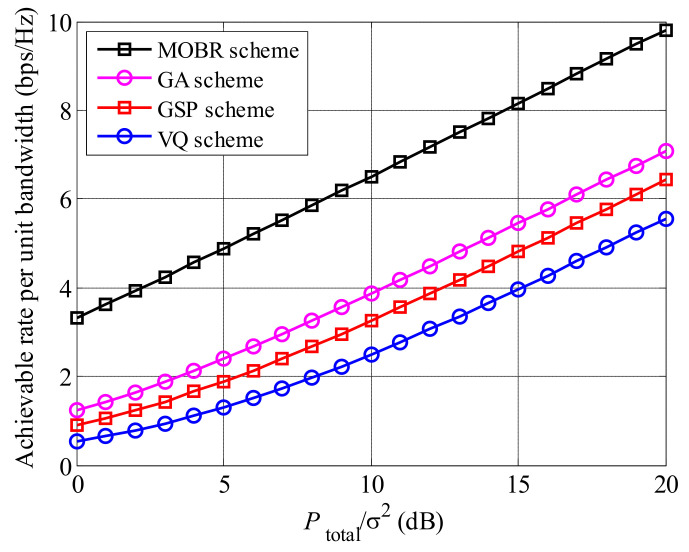
The comparisons of achievable rates among MOBR, GA, GSP, and VQ under low feedback information scenarios, where K=20 and U=20.

In Figure 6, we present the comparisons of achievable rates between MOBR and other preprocessing schemes, i.e., genetic algorithm (GA) [28], Grassmannian subspace packing (GSP) [29], and vector quantization (VQ) [30], under low feedback information condition, where K=20 and U=20. In GA, GSP, and VQ schemes, similar to the MOBR scheme, relays are introduced to enhance the received signals. The achievable rate of MOBR is the largest, followed by GA, GSP, and VQ, since MOBR can obtain the highest multi-relay selection gain and extra multi-user selection gain. Comparing GA, GSP, and VQ schemes, the GA scheme achieves the highest rate, due to the lowest distortion of the preprocessing vector.

**Figure 7:**
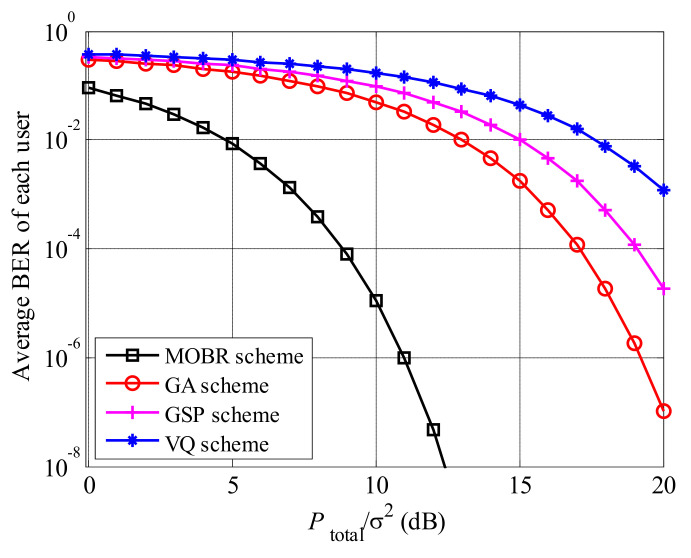
BER comparisons among MOBR, GA, GSP and VQ, where K=20 and U=20.

Figure 7 shows the BER performance of MOBR, GA, GSP, and VQ schemes, where K=20 and U=20. All curves decline with the increased $\frac{P_{\mathrm{total}}}{\sigma^{2}}$. The decline rate of MOBR is the largest, and MOBR achieves the lowest BER among these preprocessing schemes. Moreover, compared to GA scheme, MOBR achieves an extra 8 dB gain, and the reason is similar to Figure 6.

**Figure 8:**
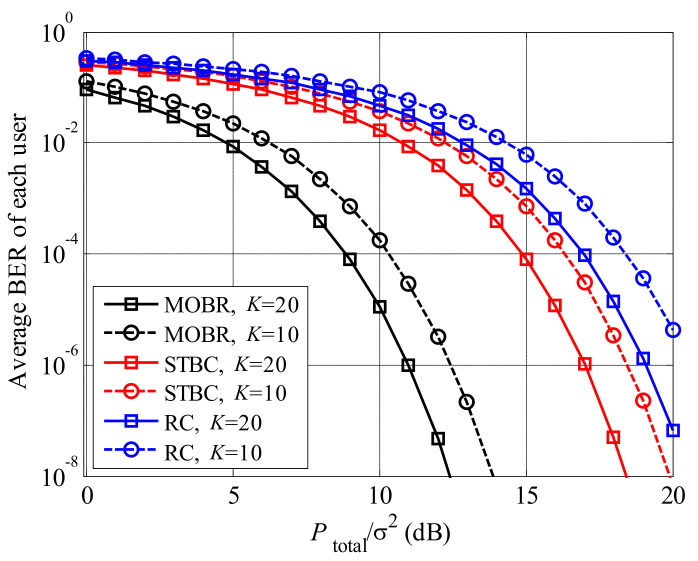
The comparisons of BERs among MOBR, STBC, and RC, where U=20.

MOBR is a kind of transmit diversity, therefore, we compare BER performance of MOBR with other two conventional transmit diversity, i.e., repetition coding (RC) [31] and space-time block coding (STBC) [32], in Figure 8. We found that the BER of MOBR is the lowest, due to the multiuser diversity gain. Comparing STBC and RC schemes, the BER performance of STBC is better than that of RC, since STBC scheme applies both space and time dimensions to improve BER performance.

**Figure 9:**
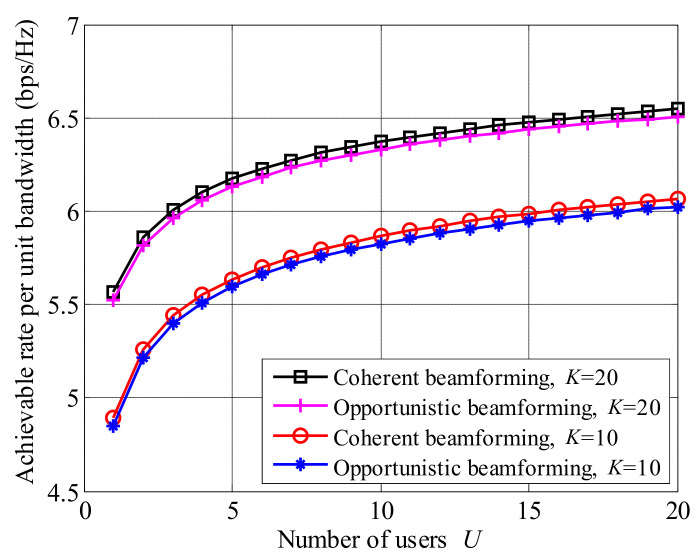
The comparison of achievable rates between coherent and opportunistic beamforming schemes, where $\frac{P_{\mathrm{total}}}{\sigma^{2}}=10$ dB and U=20.

Figure 9 presents the comparison between opportunistic beamforming and coherent beamforming [33] with relay systems, where $\frac{P_{\mathrm{total}}}{\sigma^{2}}=10$ dB and U=20. It is seen that coherent beamforming provides a higher achievable rate than opportunistic beamforming, while, the gap between coherent beamforming and opportunistic beamforming is small.

For a coherent beamforming scheme, users are required to return complete CSI, including the amplitudes and phases of all the antennas; however, for opportunistic beamforming scheme, users only feed the equivalent channels $I_{u}^{r}$ and $I_{u}^{d}$ back to BS, which reduces feedback bits. Therefore, the opportunistic beamforming scheme greatly reduces the feedback information at the cost of small achievable rate loss.

**Figure 10:**
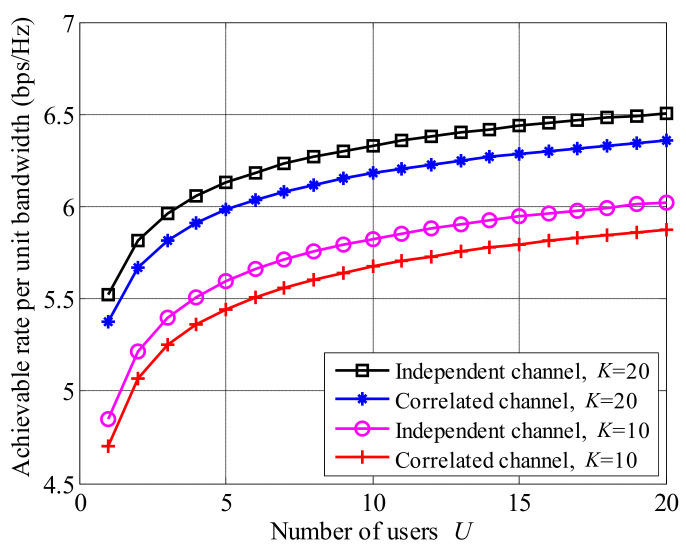
The comparison of achievable rates between independent and correlated channels, where $\frac{P_{\mathrm{total}}}{\sigma^{2}}=10$ dB and U=20.

The influences of correlated channels on the MOBR system are shown in Figure 10, where $\frac{P_{\mathrm{total}}}{\sigma^{2}}=10$ dB and U=20. The model of correlated channel is given by the case D of the 3GPP I-METRA MIMO channel model. The achievable rate of the correlated channel is smaller than that of the independent channel. The reason is that the channel coefficient fluctuation of the correlated channel is smaller than that of the independent channel, leading to lower multi-user and multi-relay selection gains.

**Figure 11:**
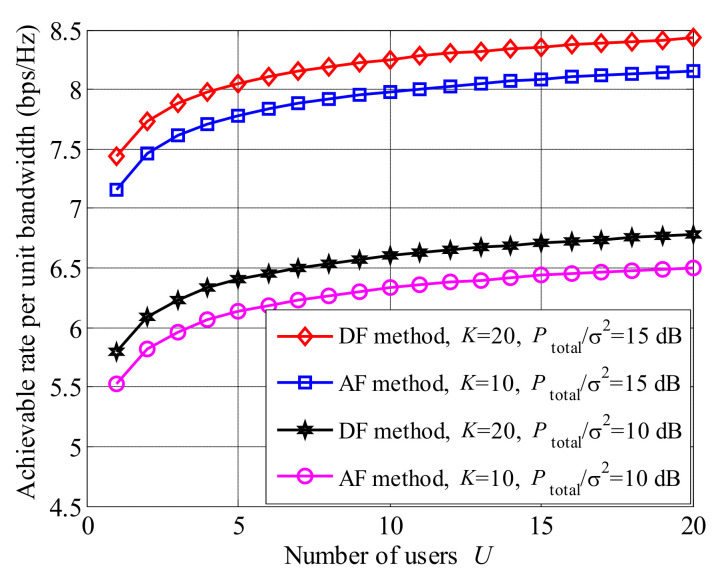
Comparison between AF and DF methods in the MOBR system.

The comparison of the achievable rate between AF and decode-and-forward (DF) methods is presented in Figure 11. It can be seen that the curves of the DF method are higher than those of the AF one, regardless of the values of both *K* and $\frac{P_{\mathrm{total}}}{\sigma^{2}}$. The reason is that both noise and signal are simultaneously amplified, when AF method is applied. However, both the complexity of AF method is lower than that of DF one. Therefore, there is a trade-off between the performances of achievable rate and complexity.

**Figure 12:**
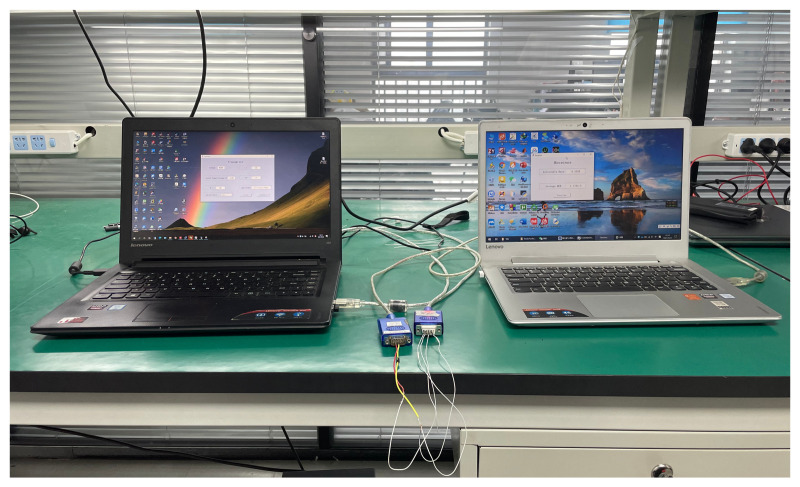
Experiment of the proposed scheme with mobile workstations.

In Figure 12, two mobile workstations are set to be the transmitter and receiver. Channel fading coefficients are generated at the transmitter. Two workstations are connected through a wire. Different parameters are considered. Then, both the achievable rate and average BER of the proposed scheme are presented in Table A1. Compared to both Figure 6 and Figure 7, the simulated and experimental results are coincident.

## 6. Conclusions

In this paper, we proposed a MOBR system, which considered both OBF and relay to improve the achievable rate. Compared to the conventional OBF systems, the achievable rate of MOBR was higher due to the multi-relay selection gain. Then, an optimization problem was formulated to maximize the achievable rate. However, the optimization problem is a NP-hard problem and is difficult to solve. To solve this problem, we divided it into two suboptimal issues and applied a joint iterative algorithm to obtain the optimal solution. Finally, we presented the simulation results, and we found that MOBR systems can obtain a high rate with low feedback information. There remain plenty of interesting topics to explore in MOBR systems, for example, multiple access techniques, the user fairness problem, and the latency issue.

## Figures and Tables

**Table 1:** The simulation parameters.

Simulation Parameters	Value
Number of transmit antennas $N_{T}$	4
AWGN variance $\sigma_{z_{u}}^{2}$, $\sigma_{z_{k}}^{2}$	$\sigma^{2}$
Ratio of transmit power to noise variance $\frac{P_{\mathrm{total}}}{\sigma^{2}}$	[0–20] (dB)
Number of relays *K*	[1–20]
Number of users *U*	[1–20]
The maximum power of relay $P_{r}^{\max}$	$\frac{P_{\mathrm{total}}}{2}$
Antenna distance in correlated channels [26]	0.5 wavelength
Power angle spectrum in correlated channels [26]	Laplace distribution
Angular spread in correlated channels [26]	$50^{o}$
Angle of arrival in correlated channels [26]	$2^{o}$

## Data Availability

Not applicable.

## Appendix A

The notations of this paper are shown in Table A2.

**Table A1:** The detailed experimental results.

Scheme	Performance	10 dB	11 dB	12 dB	13 dB	14 dB	15 dB
MOBR	Achievable rate	6.50	6.83	7.16	7.49	7.82	8.15
	Average BER	$1.12\times 10^{-5}$	$9.83\times 10^{-7}$	$4.72\times 10^{-8}$	0	0	0
GA-based	Achievable rate	3.85	4.16	4.48	4.80	5.12	5.44
	Average BER	$5.03\times 10^{-2}$	$3.27\times 10^{-2}$	$1.94\times 10^{-2}$	$1.02\times 10^{-2}$	$4.65\times 10^{-3}$	$1.87\times 10^{-3}$
GSP-based	Achievable rate	3.25	3.55	3.86	4.17	4.48	4.80
	Average BER	$9.57\times 10^{-2}$	$7.13\times 10^{-2}$	$5.01\times 10^{-2}$	$3.25\times 10^{-2}$	$1.92\times 10^{-2}$	$1.01\times 10^{-2}$
VQ-based	Achievable rate	2.49	2.77	3.05	3.35	3.65	3.96
	Average BER	$1.68\times 10^{-1}$	$1.40\times 10^{-1}$	$1.12\times 10^{-1}$	$8.71\times 10^{-2}$	$6.36\times 10^{-2}$	$4.35\times 10^{-2}$

**Table A2:** The meanings of the notations.

Notation	Meaning	Notation	Meaning
$N_{T}$	The number of transmit antennas	$y_{u}$	The received signals of users
$n_{t}$	The $n_{t}$th transmit antenna	$P_{b}$	The power allocated to BS
*U*	The number of users	$P_{k}$	The power allocated to the *k*th relay
*u*	The *u*th user	$\gamma_{u}$	The received SNR of the *u*th user
*K*	The number of relays	*R*	Achievable rate per unit bandwidth
*k*	The *k*th relay	$P_{\mathrm{total}}$	The total power
$w_{n_{t}}$	The random coefficient	$P_{k}^{\max}$	The maximum power of relay
$\sqrt{\alpha_{n_{t}}}$	The amplitude of $w_{n_{t}}$	$K_{r}$	Relay set
$\phi_{n_{t}}$	The phase of $w_{n_{t}}$	$U_{u}$	User set
w	The vector form of $w_{n_{t}}$	η,$\eta_{\mathrm{fix}}$	Power parameter
*j*	The symbol of imaginary number	$\sigma_{z_{u}}^{2}$,$\sigma_{z_{k}}^{2}$,$\sigma^{2}$	Variance
$g_{u}$	The channel from BS to user	λ	Substitution variable, equals to $\sqrt{\eta }$
$g_{n_{t},u}$	The $n_{t}$th element of $g_{u}$	$S_{\lambda }$	The feasible region of λ
$h_{k}$	The channel from BS to relay	$s_{\lambda ,\mathrm{left}}$	The left endpoint of $S_{\lambda }$
$h_{n_{t},k}$	The $n_{t}$th element of $h_{k}$	$s_{\lambda ,\mathrm{right}}$	The right endpoint of $S_{\lambda }$
$v_{k,u}$	The channel from relay to user	$\lambda_{opt}$, $\eta_{opt}$	The optimal λ and η
$x_{p,1}$,$x_{p,2}$	Pilot signals	$y_{p,1}$,$y_{p,2}$	The received signals of pilot signals
$I_{u}^{r}$	The equivalent relay channel	$i_{1}$,$i_{2}$	The labels of iteration
$I_{u}^{d}$	The equivalent direct channel	$\zeta_{p}$	The stationary point method complexity
*D*,$D_{1}$,	Objective functions	$\vartheta_{1}$,$\vartheta_{2}$	Substitution variables for simplification
$D_{2}$,${\tilde{D}}_{1}$		$\vartheta_{3}$	
